# A Comparative Study of Standardized Infinity Reference and Average Reference for EEG of Three Typical Brain States

**DOI:** 10.3389/fnins.2018.00158

**Published:** 2018-03-13

**Authors:** Gaoxing Zheng, Xiaoying Qi, Yuzhu Li, Wei Zhang, Yuguo Yu

**Affiliations:** State Key Laboratory of Medical Neurobiology, School of Life Science and Institutes of Brain Science, Center for Computational Systems Biology, Fudan University, Shanghai, China

**Keywords:** brain lateralization effect, brain functional connectivity density, phase lag index, weighted small-world network, average reference (AR), reference electrode standardization technique (REST)

## Abstract

The choice of different reference electrodes plays an important role in deciphering the functional meaning of electroencephalography (EEG) signals. In recent years, the infinity zero reference using the reference electrode standard technique (REST) has been increasingly applied, while the average reference (AR) was generally advocated as the best available reference option in previous classical EEG studies. Here, we designed EEG experiments and performed a direct comparison between the influences of REST and AR on EEG-revealed brain activity features for three typical brain behavior states (eyes-closed, eyes-open and music-listening). The analysis results revealed the following observations: (1) there is no significant difference in the alpha-wave-blocking effect during the eyes-open state compared with the eyes-closed state for both REST and AR references; (2) there was clear frontal EEG asymmetry during the resting state, and the degree of lateralization under REST was higher than that under AR; (3) the global brain functional connectivity density (FCD) and local FCD have higher values for REST than for AR under different behavior states; and (4) the value of the small-world network characteristic in the eyes-closed state is significantly (in full, alpha, beta and gamma frequency bands) higher than that in the eyes-open state, and the small-world effect under the REST reference is higher than that under AR. In addition, the music-listening state has a higher small-world network effect than the eyes-closed state. The above results suggest that typical EEG features might be more clearly presented by applying the REST reference than by applying AR when using a 64-channel recording.

## Introduction

Electroencephalography (EEG) records the mean electrical activity of the brain in different sites of the head (Berger, 1929). The signals reflect the total electronic potential projected from large pools of the extracellular current flows of tens of thousands of neurons under the electrode recording site. It is one of the most important tools in clinical neurophysiology and is also applied in human cognitive research because of its high temporal resolution and noninvasive safety feature (Cohen, 2017). However, to date, EEG analysis, especially in clinical diagnostic examinations, still relies primarily on visual inspection, and there is a lack of standard analysis approaches and standard references (Nunez et al., 1997) to help decipher the functional meaning of the scalp-recorded EEG. To evaluate the accurate characteristics of EEG (including electrode polarity, scalp area, frequency spectrum, latency and voltage amplitude), it is usually necessary to use an inactive electrode as a reference, making the selection of a reference electrode particularly relevant and critical (Yao, 2001). Electrode reference methods commonly used in EEG studies include the average reference (AR), the linked mastoid reference (LM), the vertex reference (Cz), and the infinity zero reference. Previous studies using model simulation have revealed that non-zero references, including AR, LM and Cz, can result in distortion of scalp power spectral distribution, changes in the potential of electrodes and differences in network configurations (Yao et al., 2005, 2007). Yao et al. have developed a theoretical approach to propose an infinity zero reference to minimize errors by using the reference electrode standardization technique (REST) (Yao, 2001; Dong et al., 2017). REST is a method that approximately converts a record that uses a point or mean potential on the scalp as a reference point to a spatial infinity point as a reference position. The physical basis of this operation is that the potentials before and after conversion are all derived from the actual neural electrical activity source in the brain (Hu et al., 2018). Although different reference electrodes have been investigated in many studies (Ferree, 2006; Nunez and Srinivasan, 2006; Huang et al., 2017; Lei and Liao, 2017; Qin et al., 2017), most of them are based on model simulated data; it is still unclear what the accurate estimation of the differences is for real EEG recordings in various experimental conditions.

While different cognitive tasks involve different brain regions, more researchers are focusing on the changes of brain functional networks (Bullmore and Sporns, 2009; Van Den Heuvel and Pol, 2010). Studying the effect of reference electrodes on brain functional networks may be critically important to access functional meanings of EEG recordings.

Researchers have found that biological networks generally have the characteristics of small-world network properties, that is, the network has a large clustering coefficient and a short characteristic path length (Watts and Strogatz, 1998; Strogatz, 2001). Shi et al. found in electrophysiological recordings of primary visual cortex in mice that their coding properties have small-world structural properties (Shi et al., 2015). He et al. applied the reciprocal of global efficiency instead of the traditional characteristic path length in the study of magnetic resonance brain network characteristics, effectively avoiding the problem of isolated points of the network (Liao et al., 2017). Wu et al. found that music perception shows an enhancement of small-world network organizations in brain functional connectivity (Wu et al., 2012, 2013).

The aim of this study is to directly evaluate the choice influence of AR and REST on the properties of brain lateralization effect, brain functional connectivity density and small-world network characteristics in the analysis of recorded EEG signals during resting and music listening states.

## Materials and methods

### Participants

The experiment collected EEG data from 38 healthy subjects (mean age 21.55 years, standard deviation (SD) 3.70; right-handed; 18 females and 20 males). All participants signed an informed consent form before the experiment. This project was approved by the Ethics Committee of Fudan University. Most of the subjects had no professional music training except for one student who had previously received piano training. None of the participants took medication within the first 2 weeks of the experiment or had a history of potential mental illness.

### Experimental procedures and EEG recording

**Figure 1:**
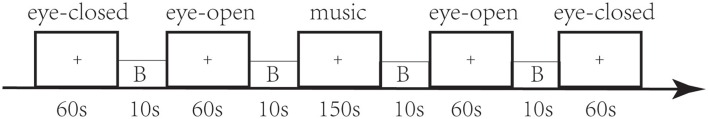
Experimental protocol used in our study. All subjects were asked to keep their eyes closed for 1 min without thinking about anything, and after a short break (B = Break), they kept their eyes fixed on the “+” in the middle of the screen for 1 min. After 2 min in the resting state, there was two and half minutes of music playing; subjects were asked to keep their eyes closed and listen quietly without thinking about anything. Next, there was 1 min with eyes open, followed by 1 min of eyes closed, and then the experiment was complete.

EEG data were recorded using the actiCHamp 64-channel EEG system from Brian Products, and the sampling rate was 1,000 Hz. Using Cz as a reference, all the other electrodes were placed symmetrically on both sides of the brain. The impedance of all electrodes was maintained below 10 kΩ during the experiment. The entire experimental paradigm was completed in the E-Prime Pro 2.0 software environment. At the beginning of the experiment, the participants were asked to keep their eyes closed for 1 min, and then to keep their eyes opened and fixed on the screen for 1 min. After a short break, they listened to the selected music for approximately two and a half minutes with their eyes closed. Next, participants were asked to keep their eyes open for 1 min and then keep their eyes closed for another minute in a quiet condition. The detailed experimental procedure is shown in Figure 1.

In the experimental protocol, the experiment consists of 60 s eyes closed, 60 s eyes open, 150 s music, 60 s eyes closed and 60 s eyes open with EEG recordings. In the data preprocessing, the 10 s before and after each state (eyes closed, eyes open or music state) are discarded in order to reduce the error caused by the moment of state change, and the remaining EEG data were used for analysis. We choose the first period of eyes closed and eyes open because the brain activity in the second period may be influenced by the previous music listening.

### Data preprocessing

We used MATLAB R2015b to process the EEG data. EEG data were divided into different segments according to the event marker, and each segment was filtered to be in a frequency range of 0.5–100 Hz. Meanwhile, the 50 Hz frequency interference was removed by using a notch filter. Data preprocessing was performed using the EEGLAB toolbox (Delorme and Makeig, 2004) to remove artifacts based on the ICA algorithm. The plugin ADJUST toolbox identified the artifact components for removal. All preprocessing was kept equivalent except for choosing the two different reference methods. We aimed to compare the difference in the data processing when using the average reference (AR) and REST (Yao, 2001) to evaluate the influences of reference selection on the analysis. We applied the MATLAB ANOVA toolbox to examine the differences in using these references.

In traditional studies, a large amount of experimental analysis advocated for the average reference as the best available reference option during EEG recording and analysis. As the number of electrodes is large enough to cover the whole brain, it is widely believed that the average potential of all the electrodes can be regarded as approaching the zero-potential point (Bertrand et al., 1985). However, a more recent paper (Yao, 2017) noted that such a zero integral assumption has been proven only for a spherical surface. Indeed, the potential integral over the surface of a dipole in a volume conductor may not be zero, depending on the shape variances of the head-shape conductor and the orientation changes of the head conductor with electronic signals flowing through it. Thus, the average reference might not be a theoretical “gold standard” reference. In particular, in the case of small numbers of electrodes in the EEG recording, AR may provide a large biased estimate of reference-independent potentials. To entirely resolve the problems involved in using body surface points for referencing, a reference with neutral potential is required. Theoretically, a point at infinity is far from brain sources and has an ideally neutral potential. Yao et al. (Yao, 2001; Dong et al., 2017) proposed a reference electrode standardization technique (REST) to mathematically transform the actual multi-channel EEG data recorded with a scalp point reference to recordings referenced at infinity reference (the Matlab toolbox for REST transformation can be downloaded at www.neuro.uestc.edu.cn/rest) (Dong et al., 2017). This technique transforms the EEG potentials referenced at AR into potentials referenced to an infinity point, i.e., a zero-potential point. For both the AR and REST methods, this experiment has used the same placement of the electrodes. Prior to the REST transformation, the real electrode coordinates and the scalp EEG recordings with average reference (AR) should be obtained first from the experiment method as well as EEGLAB pre-processing process. A head model and equivalent source model in the REST toolbox are then implemented to calculate the new channel data, referenced at the infinity reference.

To verify the validity of EEG data after preprocessing, the power spectrum at resting state were analyzed. In general, the alpha rhythm is the prominent EEG wave pattern of an adult who is awake but relaxed with eyes closed. Each region of the brain has a characteristic alpha rhythm, while alpha waves of larger amplitude are generally observed in the frontal and occipital regions. The amplitudes of alpha waves diminish when subjects open their eyes. This is called the alpha-blocking effect. To quantify under which reference condition the alpha-blocking effect is distinguished, we calculated the reduction of total alpha power $\bar{\Delta P_{\alpha }}$ in the frequency range of 8–12 Hz for each channel and then averaged over 64 channels.

(1) $$\begin{array}{l} \bar{\Delta P_{\alpha }}=\frac{1}{64}\sum_{i\;=\;1}^{64}\overset{12}{\underset{f\;=\;8}{\sum }}[\left(log_{10}\left(P_{close}(f)\right)-log_{10}\left(P_{open}(f)\right)\right)\\ \;/\left(log_{10}P_{close}(f)\right)] \end{array}$$

where *P*_*close*_*(f)* and *P*_*open*_*(f)* represent power spectrum of recorded EEG signals.

All the alpha-blocking effects of the 38 subjects were calculated to compare the difference between REST and AR.

### Brain lateralization analysis

The human brain has specific task division in the process of information processing, and different brain regions have their own preferences in handling tasks LeMay, 1977). Studies have indicated that there is typical left brain lateralization in the language region (e.g., Broca Area and Wernicke Area) and in the default network (e.g., lateral intraparietal sulcus, anterior insula, area MT, and frontal eye fields), whereas there was significant right-lateralization of the attention control network (Stephan et al., 2003; Nielsen et al., 2013). Researchers have found that the degree of lateralization of the brain is significantly different between patients with autism spectrum disorders or schizophrenia and normal people, and the study of brain lateralization may be important for understanding the mechanism of neurological disease (Chance et al., 2008; Kleinhans et al., 2008). In this paper, we analyzed the lateralization of the prefrontal cortex brain regions under the resting states. We used the logarithm of the power spectrum in the alpha band to obtain the lateralization index (LI), calculated as LI =(Pleft-Pright)/(Pleft+Pright), to quantify the lateralization effect. Our aim was to evaluate the difference between AR and REST from the perspective of the brain lateralization effect.

### Phase lag index

We built up networks of EEG brain functions in an attempt to determine the effect of different reference choices on analysis results under different behavior states. To construct the brain functional network, we defined the relationship between the two electrodes as the state of connection among the networks. The phase lag index (PLI) was calculated to evaluate the connection characteristics between each pair of electrodes (Wu et al., 2013; Hardmeier et al., 2014). PLI indeed quantifies the consistency of phase synchronizations between two electrodes (Stam et al., 2007). For any segment of EEG signal *x*(*t*), its phase ϕ(*t*) can be calculated by a Hilbert transform:

(2) $$\phi \left(t\right)=\arctan\frac{x_{H}\;\left(t\right)}{x\;\left(t\right)}$$

where *x*_*H*_(*t*) is the imaginary part of the signal after the Hilbert transform. For two signals, *x*_*a*_ (*t*)and*x*_*b*_ (*t*), the phase difference can be expressed as Equation (3):

(3) $$\varphi_{ab}\left(t\right)=\phi_{a}\left(t\right)-\phi_{b}\left(t\right)$$

The PLI is then calculated as:

(4) $$PLI_{ab}=\left|\frac{1}{N}\sum_{n=0}^{N-1}sign\left(\varphi_{ab}\left(n\right)\right)\right|$$

where *sign*(φ_*ab*_(*n*)) returns 1 when φ_*ab*_(*n*) > 0 and returns −1 when φ_*ab*_(*n*) < 0. The symbol N is the number of sampling points. The stronger the nonzero phase locking is, the larger PLI and stronger phase synchronization will be. The PLI value is restricted in the range of 0~1, and PLI = 0 means no coupling (or coupling with a relative phase that encircled 0 modπ, which is likely to result from volume conduction). PLI = 1 indicates the condition of strict phase locking at a constant nonzero phase lag.

### Network analysis

To study brain functional connectivity in different states, global brain functional connectivity density (GFCD) and local brain functional connectivity density (LFCD) (Tomasi and Volkow, 2010, 2011) were adopted. GFCD characterizes the connection between one electrode and the others among the whole brain, and LFCD can characterize the connectivity relationship between local scalp recording channel networks. To access the LFCD of the preprocessed EEG activities, there are two parameters to calculate: the threshold Tc determines the significant correlations between two channels, and the local cluster radius determines the size of the local scalp recording networks. Taking into account that a lower Tc may increase the false positive rate and become larger with lower sensitivity, we computed Tc based on the small network, and the local cluster radius was set to 5 cm. We first computed the PLI for all pairs of EEG channels. The PLI values were considered significant if they were larger than the Tc, and the number of significant PLI values per channel was computed as LFCD values. The preprocessing and post-processing steps of GFCD are similar to LFCD, except with the local cluster restrictions.

### Weighted small-world network

This network consists of a series of points and the edges that connect these points. Above, we used the PLI to describe the functional connection strength of each pair of electrodes, and then we could construct a brain network using the PLI matrix (Stam et al., 2007; Hardmeier et al., 2014). Here, we set the thresholds (start is 0.01, step is 0.01, end is 0.99) to construct different brain functional networks using different thresholds (Liu et al., 2008; Rubinov and Sporns, 2010). We investigate the clustering coefficients and characteristic path lengths to compare the difference of network characteristics under different reference electrodes. In this paper, we choose a weighted network, as it can more truly reflect the functional connections of the brain network, and we define the PLI as the weight between the two vertexes when the value is greater than the threshold.

For a network, the clustering coefficient of the vertex *i* characterizes the proportion of connections among their neighbors. It can be calculated as (Onnela et al., 2005)

(5) $$C_{i}=\frac{\sum_{j\neq i}\sum_{\begin{array}{c} k\neq i\\ k\neq j \end{array}}w_{ij}w_{ik}w_{jk}}{\sum_{j\neq i}\sum_{\begin{array}{c} k\neq i\\ k\neq j \end{array}}w_{ij}w_{ik}}$$

Where *w*_*ij*_ is the edge weight (i.e., PLI value) between vertex *i* and *j*, when the PLI is larger than a given threshold, otherwise, *w* equals 0.

The clustering coefficient of the whole network can be obtained by averaging the clustering coefficient over all vertexes.

(6) $$C=\frac{1}{N}\sum_{i\;=\;1}^{N}C_{i}$$

where *N* represents the number of nodes in the network. The traditional characteristic path length is defined as the average of the distance between any two nodes; such a definition will bring outliers in the network. We applied the global efficiency to characterize the path length of a weighted network, in which the length of an edge equals the reciprocal of the edge weight when it does not equal 0, while it returns infinity if the weight equals 0. The shortest path length between node *i* and node *j* was defined as*d*_*ij*_, and the characteristic path length of the entire network can be defined as the reciprocal of global efficiency (Latora and Marchiori, 2001).

(7) $$L=\frac{1}{E}=\frac{1}{\left(1/N\left(N-1\right)\right)\sum_{i\;=\;1}^{N}\sum_{\begin{array}{c} j\;=\;1\\ j\;\neq \;i \end{array}}^{N}d_{ij}^{-1}}$$

where *N* represents the number of nodes in the network. We calculated the clustering coefficient divided by the characteristic path length as the small-world property (SW). SW characterizes the efficiency of network information transmission and the network connection cluster size (Bassett and Bullmore, 2017). Then, small-world effects under different states were calculated to evaluate the influences of reference choice.

(8) $$SW=\frac{C}{L}$$

## Results

### Alpha-blocking results

**Figure 2:**
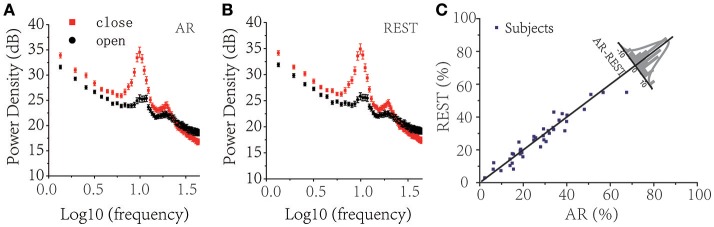
Resting EEG Power spectrum analysis between eyes-closed (red square solid points) and eyes-open (black round solid dots) under different reference choices. **(A)** AR, **(B)** REST. **(C)** Difference of alpha-blocking rate (characterized as $\bar{\Delta P_{\alpha }}$ under alpha band, 8–12 Hz, Pclose is the summation of logarithm of eyes-closed power spectrum and Popen is the summation of the logarithm of the eyes-open power spectrum) of 38 subjects (average all electrodes) at different reference electrodes (AR & REST), in which 19 of 38 subjects showed that REST performed better than AR.

The human alpha rhythm is generally defined as neural electronic oscillations with a frequency of 8–12 Hz and is normally recorded as sinusoidal waves with larger amplitudes over posterior head regions, present in approximately 95% of healthy adults, especially during wakeful relaxation with an eyes-closed resting state (Berger, 1929). The alpha waves are temporarily blocked or largely reduced by in the influx of light input during the eyes-open state or other afferent stimuli (see Figures 2A,B). Pfurtscheller (Pfurtscheller and Klimesch, 1990) conceived the stimulus-induced alpha-blocking phenomenon as desynchronized neural population activities during active stimuli. Smith et al. (1998) have observed unilateral alpha rhythm to eye opening and closing, that is, right-sided alpha activity appears with the left eye closed, while left-sided alpha activity occurs with the right eye closed. Alpha-blocking occurs with the opening of both eyes. Therefore, in our study, we could use the alpha-blocking effect with eyes open compared with eyes closed to monitor the stability of experimental recording processes.

We first quantified the alpha-blocking effect of the eyes-open condition compared with the eyes-closed condition to verify the validity of the data (see Figures 2A,B). By calculating the difference in the logarithm of alpha wave power spectrum between eyes-closed and eyes-open as the signal-to-noise ratio (SNR), we observed that 19 out of 38 subjects had a larger alpha-blocking effect with REST as a reference than with AR (Figure 2C). Matlab ANOVA Toolbox was applied to test the statistical significance between AR and REST of the alpha-blocking effect, and ANOVA1 analysis showed there was no significant difference [*F*_(1, 74)_ = 0.02, *P* = 0.8842 > 0.05] between AR and REST in the alpha-blocking effect. The two methods were almost equivalent to each other in the analysis of the alpha-blocking effect of the eyes-open condition.

### Brain lateralization effect

**Figure 3:**
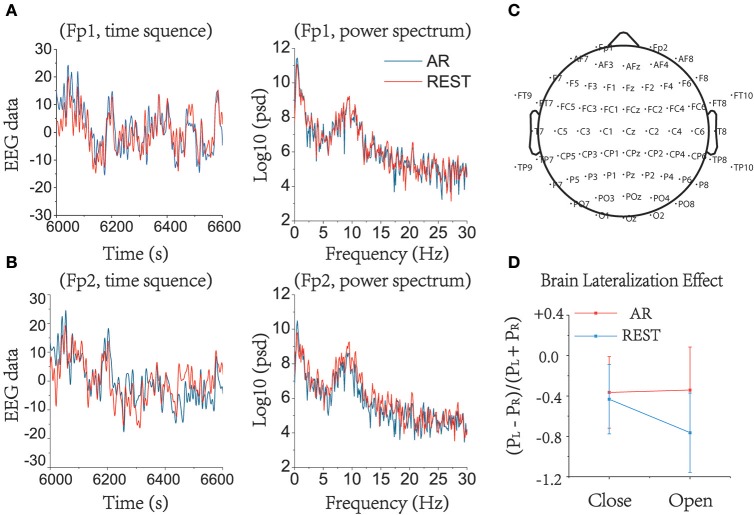
Brain lateralization effect in prefrontal area (Left: Fp1, AF7, AF3, F7, F5, F3, F1; Right: FP2, AF8, AF4, F8, F6, F4, F2) in alpha band under resting state. EEG raw data and power spectrum under eyes-closed when using AR and REST as a reference in **(A)** Fp1. **(B)** Fp2. It can be seen that AR and REST can result in differences when preprocessing the data. **(C)** The array of 64 electrodes used in this study. **(D)** Brain lateralization effect in alpha band between left prefrontal area and right prefrontal area, characterized as (PL – PR)/(PL + PR). The degree of lateralization under REST was higher than under AR.

Figures 3A,B depicts the effects on the EEG data preprocessing and the power spectrum analysis under AR and REST. REST appeared to make the signal smoother than AR. Left and right lateral cerebral hemispheric effects at resting state in the prefrontal area please see Figure 3C (Left: Fp1, AF7, AF3, F7, F5, F3, F1; Right: FP2, AF8, AF4, F8, F6, F4, F2) showed that there was indeed more alpha wave activity in the right brain than in the left. Additionally, REST had a greater degree of lateralization than AR (Figure 3D). The ANOVA1 indicated that there was no significant difference between AR and REST, whether in the eyes-open state [F_(1, 74)_ = 1.55, *P* = 0.2164 > 0.05] or in the eyes-closed state [F_(1, 74)_ = 3.11, *P* = 0.0819 > 0.05]. The statistical results showed that the different references may induce a difference in brain lateralization. However, the differences are not statistically significant between AR and REST.

### Brain functional connectivity density results

**Figure 4:**
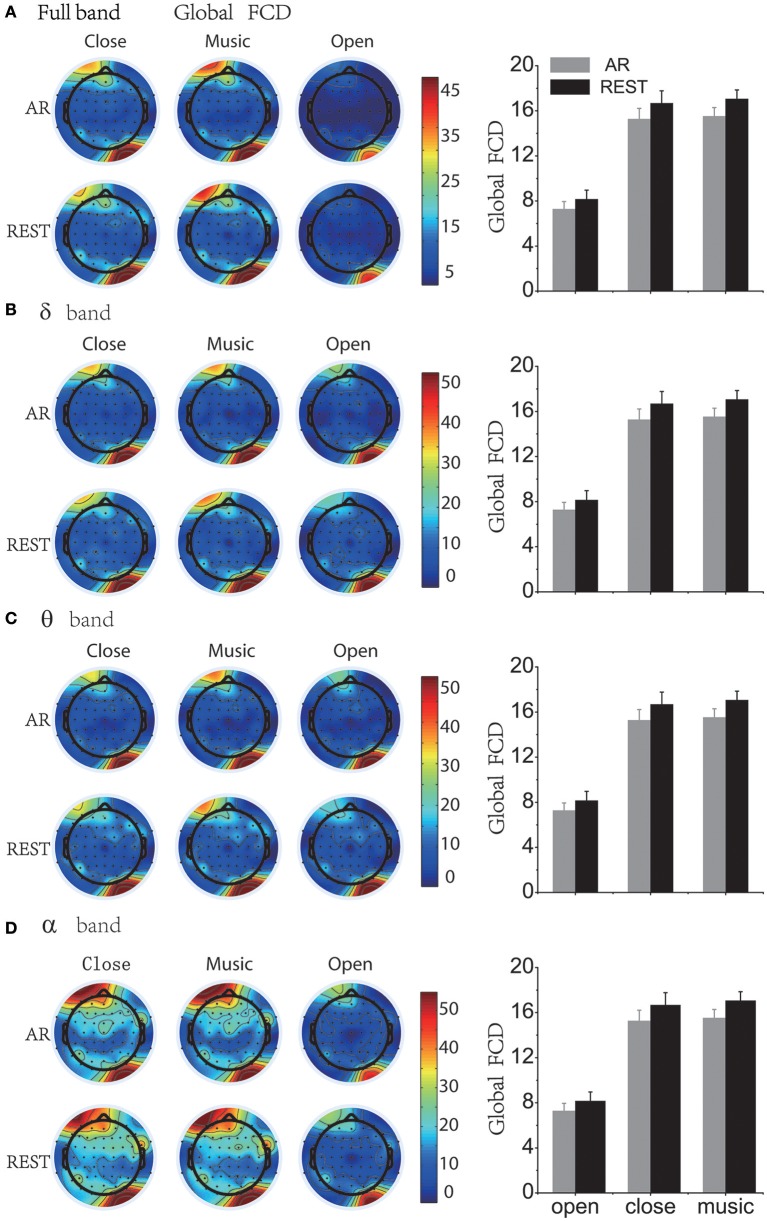
Global brain functional connectivity density (Global FCD) topography and average of Global FCD under AR and REST reference. “AR” = average reference and “REST” = reference electrode standardization technique, that is, infinity reference. **(A)** Full band. **(B)** Delta band. **(C)** Theta band. **(D)** Alpha band. For high-frequency bands such as beta and gamma bands, please see the Supplementary Materials.

**Figure 5:**
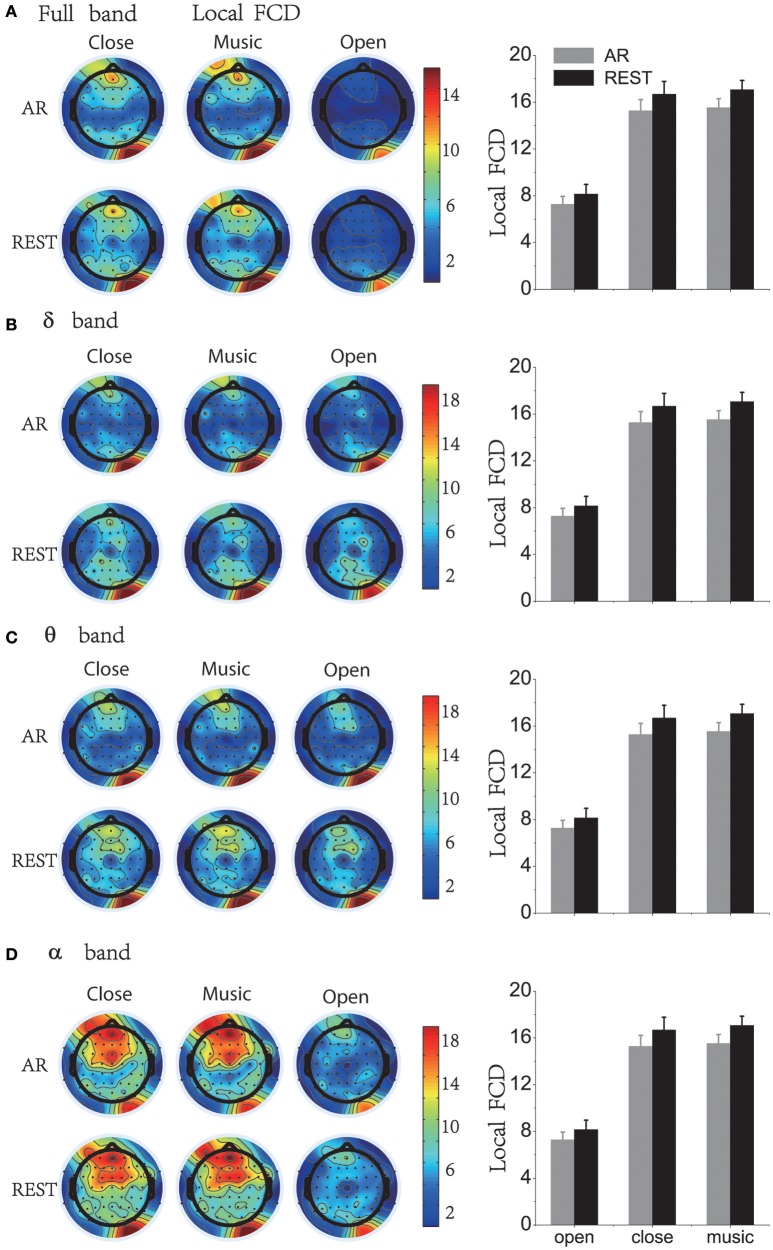
Local brain functional connectivity density (Local FCD) topography and average of Global FCD under AR and REST reference. “AR” = average reference and “REST” = reference electrode standardization technique, that is, infinity reference. **(A)** Full band. **(B)** Delta band. **(C)** Theta band. **(D)** Alpha band. For high-frequency bands such as beta and gamma bands, please see the Supplementary Materials.

The result of global functional connectivity density (Global FCD) showed that brain functional connectivity is stronger in the eyes-closed state than in the eyes-open state (*p* < 0.01, ANOVA1 analysis). The distribution of FCD under the eyes-closed state was larger in the left frontal area than other regions, indicating that the strength of the brain networks was much more activated at rest, especially in the alpha band. The distinction between eyes-closed and music-listening is obvious in full bands and low frequencies (e.g., theta and delta bands), illustrating that listening to music can activate slow-wave brain rhythms in frontal lobes. The comparison between REST and AR indicated that brain functional connectivity density under the REST condition has a larger value than the AR condition in the eyes-open state in all frequency bands for both global FCD (see Figure 4) and local FCD (see Figure 5). The averaged global and local FCD values under the eyes-closed condition are significantly higher than those under the eyes-open condition in full, alpha, beta and gamma frequency bands (Matlab ANOVA1, *p* < 0.01), while the music-listening condition is almost equivalent to the eyes-closed condition (see right panels of Figures 4A–D, 5A–D). The significant difference analysis is provided in the Supplementary Material for the right sub-panels in Figures 4, 5. Table S2 lists the statistically significant results of Figure 4 and Figure S2. Table S3 lists the statistically significant results of Figure 5 and Figure S3.

### Brain functional network results

**Figure 6:**
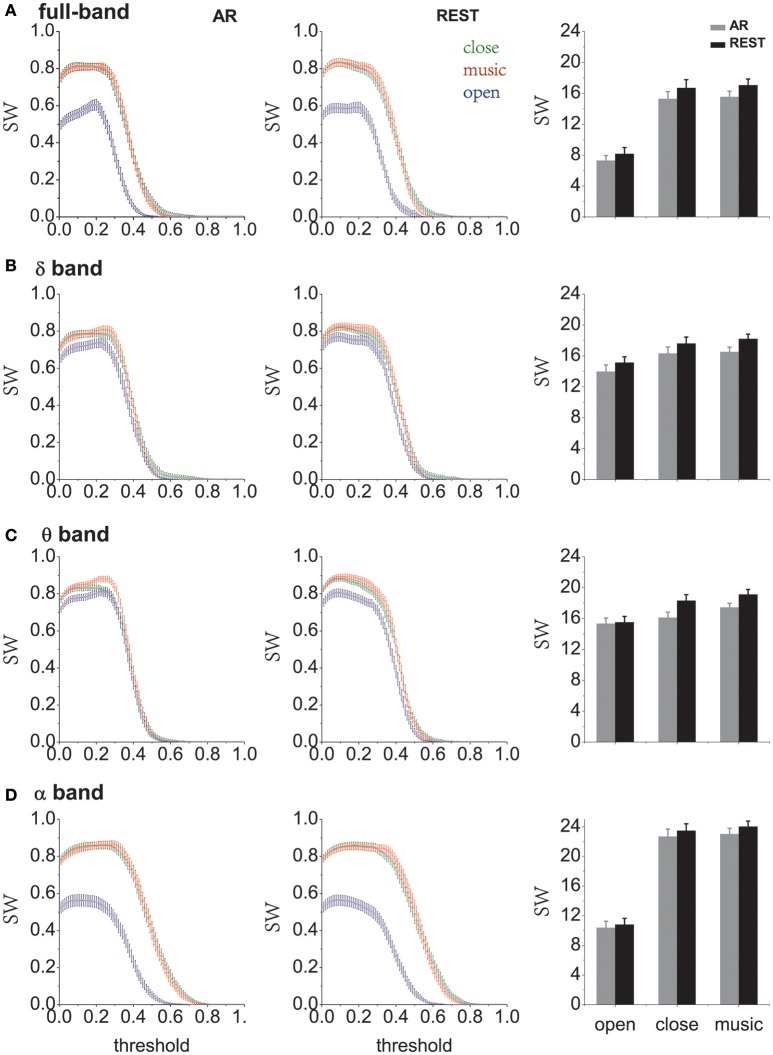
The small-world characteristics in different states (eyes-open, eyes-closed and music) under different reference electrodes. “SW” = small-world characteristics. “AR” = average reference and “REST” = reference electrode standardization technique, that is, infinity reference. **(A)** Full band. **(B)** Delta band. **(C)** Theta band. **(D)** Alpha band. For high-frequency bands such as beta and gamma bands, please see the Supplementary Materials.

When comparing different resting states, we found that the values of small-world characteristics in the eyes-closed condition are more significant than those of the eyes-open condition (Matlab ANOVA1 analysis, *p* < 0.01). Furthermore, the small-world values for all the different states under the REST reference are observed to be higher than those for the AR condition (Figure 6). In addition, the results of different frequency band analysis indicated that brain have a larger small-world network property in the eyes-closed state than in the eyes-open state (Figure 6). We calculated the sum of the small-world values within the significant threshold range (threshold from 0.2 to 0.5) and found that the small-world effect showed the following trend: SWmusic > SWclose > SWopen in full- and low-frequency bands (including delta, theta and alpha bands). Additionally, the difference between eyes-closed and eyes-open was significant in full, alpha, beta and gamma frequency bands (*p* < 0.01, ANOVA1), and the music-listing state and eyes-closed state have no significant difference in all bands (*p* > 0.05, ANOVA1) (see right panel of Figures 6A–D, and Figure S1). Please also see Table S1 and Figure S4 for the statistically significant results for Figure 6.

## Discussion

In recent years there have been an increasing number of theoretical and experimental studies suggesting the superior merits of REST. Over the years, many researchers have provided evidence and advice on the choice of different reference electrodes. Simulation and experimental ERP studies with a visual oddball paradigm indicated that AR and LM (Linked Mastoids) may change the amplitude of P300 and distort the CM trajectory and its instantaneous velocity, while REST continues to provide objective results (Qin et al., 2017). A large-scale brain network simulation analysis compared REST with Fz, Oz, LM and AR and showed that the relative errors followed the pattern REST < AR < LM < (Fz, Oz), which indicated that REST was a potentially preferable reference (Lei and Liao, 2017). In addition, high electrode density helps to lower the re-referencing reconstruction errors for both AR and REST (Liu et al., 2015). A functional connectivity graph study showed that REST not only achieved excellent performance for superficial and radial sources but also performed stable and robust variable source locations and orientations, while AR performed stably only when there was no upward component in the sources' orientation (Huang et al., 2017). With high-density electrode coverage, AR may approach the true reference method, as it averages the signals and noise of all the electrodes (Ferree, 2006; Nunez and Srinivasan, 2006).

This study aimed to evaluate the differences between AR and REST from the perspective of power spectrum density, the brain lateralization effect and brain functional network based on real recorded EEG data. During the eyes-open resting condition, the typical alpha-blocking effect could be detected from the EEG recordings. Our results show that half of the 38 subjects were observed to have larger alpha-blocking effects with the REST reference than with AR when using 64 channel recordings. This suggests that in certain situations when using a relatively large number of EEG recording channels, the differences between AR and REST analysis results might be difficult to detect. In this case, neither REST nor AR can be regarded as a “gold standard.” Furthermore, we observed a clear right-lateralized phenomenon in the prefrontal area at resting state, which is consistent with previous reports (Nielsen et al., 2013). Additionally, the brain lateralization effect under REST was more significant than that under AR (Figure 3D). Brain functional network results indicated that small-world property values under different states showed the following tendency: music > close > open (Figure 6). The results of the music state may be instructive for the study of music to improve brain function. After counting the small-world property values in the significant threshold, it was found that REST was generally more effective than AR (see right panel of Figures 6A–D). As indicated more recently by Yao (2017), because the shape of the human head is not a perfect spherical surface and its dipole orientation is not theoretically perfect, using the average value over all recording sites as an AR reference might be far from a zero reference. For these reasons, REST may provide better analysis results than AR to reveal brain functional network properties (Qin et al., 2010).

### Reference influence on brain lateralization effect

Resting anterior EEG asymmetry in the alpha (8–12 Hz) band is known as a potential bio-marker of emotional predisposition (Tomarken et al., 1992). Generally, positive emotion has been associated with left-frontal activation (i.e., less alpha wave in left than right brain regions, as alpha activity is taken as an inverse index of brain activation), while negative emotion has been associated with higher activation of the right frontal regions (Henriques and Davidson, 1990). The 38 subjects employed here were all from Fudan University, and none had depressive or anxiety symptoms measured by subscales of the SCL-90 (Derogatis and Cleary, 1977). Our results demonstrated that most of the subjects had more distinct right-lateralized phenomenon in the prefrontal area, i.e., more alpha wave in right brain regions especially, under the REST condition than under AR. Perhaps EEG analysis under the REST condition preserves more information than that under the AR condition due to averaging. It should be noted that this study only compared the degree of lateralization under resting states and did not conduct further studies on emotion-related effects. In addition, the effects of lateralization can only reflect the differences between the left and right sides and cannot reveal the entire brain functional connectivity in different states. The brain functional network is critical for us to understand how the brain processes information efficiently.

### Reference influence on brain functional network

Neuroscientists are becoming increasingly concerned with the study of the brain functional network; however, the choice of reference electrodes may lead to relatively large deviations of this network. We studied the brain functional network characteristics of subjects in different states and found that in the resting state, the average properties of weighted small-world at eyes-closed were significantly higher than those at eyes-open (*p* < 0.01, ANOVA1 analysis). The difference between eyes-closed and eyes-open in all bands shows that REST was higher than those at average reference (Figure 6). Although our results have indicated that in most situations the estimated measures using REST were larger than those using AR, this does not mean that REST is a better reference than AR unless further solid evidence becomes available.

In this study, the measurement of brain functional connectivity may be problematic, although using the electrodes as nodes of the network is primarily used in this study. EEG signals recorded on the scalp consist of synaptic electrical activity from neurons in the cerebral cortex. The ideal method of brain functional connectivity analysis is to locate the signal source of cognitive activity and then analyze the flow of information among different sources. However, the source analysis of EEG recordings is not completely accurate, and the source location is under dispute. First, EEG is not reliable for source localization because of its low spatial resolution. Second, due to the volume conduction effect, the cerebrospinal fluid, skull, and scalp cause the electrical current generated by ensembles of neurons deep inside the cortex to diffuse spatially before reaching the scalp electrodes. Thus, each recording from an electrode on the scalp is not necessarily generated by a source below that electrode. This is the typical volume conductor problem which produces relatively large zero-lag correlation among recording electrodes. The traditional network analysis cross-correlation and phase coherence are seriously distorted by the volume conduction effect (Christodoulakis et al., 2013). However, the volume conductor effect could be largely avoided in functional network re-construction by using the phase lag index, which uses phase information of time sequences of different channels to construct the network correlations (Peraza et al., 2012). In this way, the reconstructed brain functional network might reflect real spatio-temporal relations among different scalp recording channels. Even when this works well, each individual recording channel signal is still a mixture derived from underlying neuronal pool resources within the deep cortex. A better method would be to use magnetic resonance imaging (MRI) to locate the source and then use EEG to explore the flow of information among different sources. In the future we will combine EEG and fMRI to locate the sources of different cognitive tasks to reveal useful information.

## Conclusions

In this study, we evaluated the difference between the average reference and the infinity reference from the perspective of alpha-blocking, brain lateralization effect, functional connectivity density and weighted small-world network characteristics. Alpha-blocking results indicated REST and AR had no significant difference. The brain lateralization effect results indicated that infinity reference had a greater lateralization effect than the average reference. The network results showed that small-world network parameters in the REST condition were larger than that of AR for all frequency bands. These distinct results deserve further exploration. In sum, the choice of reference electrode can introduce some bias into analysis results. Our results showed that in most cases, the typical features evaluated here were more clearly presented when EEG was referenced by REST. AR might smear these intrinsic effects slightly. However, until verified by further studies the application of both REST and AR during analysis might be a reasonable way to validate analysis-derived measurements.

## Ethics statement

This study was approved and supervised by the Ethics Committee of the School of Life Sciences at Fudan University (No.290). All participants signed written informed consent.

## Author contributions

YY supervised the research; GZ, WZ, and YY designed the research; GZ, WZ, YL, and XQ performed the research, and GZ and YY wrote the paper. All authors reviewed the manuscript.

### Conflict of interest statement

The authors declare that the research was conducted in the absence of any commercial or financial relationships that could be construed as a potential conflict of interest.
