# Supplementary material for: A Comparative Study of Standardized Infinity Reference and Average Reference for EEG of Three Typical Brain States
Source: Front Neurosci. 2018 Mar 13;12:158. doi: 10.3389/fnins.2018.00158 (PMC5859052; doi:10.3389/fnins.2018.00158)
Supplement: Supplementary file 1 [file DataSheet1.pdf]

Supplementary materials

Figure S1

The small-world characteristics in different states (eyes-open, eyes-closed and music) under different references in high-frequency band situations. **(A)** Beta band. **(B)** Gamma band. ‘SW’ = small-world characteristics. ‘AR’ = average reference and ‘REST’ = reference electrode standardization technique, that is, infinity reference. It can be seen that the SW under eyes-open was much lower than that under eyes-closed and music-listening, while the music-listening state and the eyes-closed state showed no significant difference. The statistical significance can be seen in Figure S4 and Table S1.

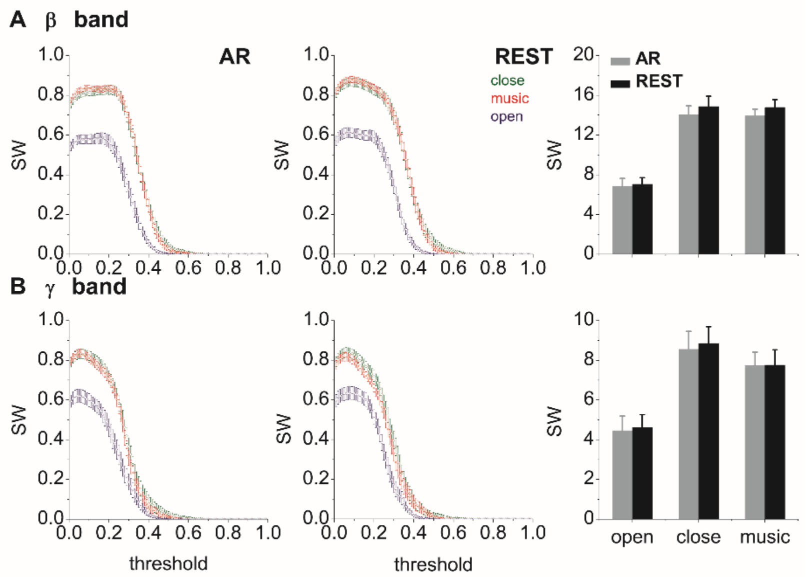

Figure S2

Global brain functional connectivity density (Global FCD) topography and average of Global FCD under AR and REST reference in high-frequency band situations. **(A)** Beta band. **(B)** Gamma band. It can be seen that there was left frontal brain activity during eyes-open, and the alpha wave disappeared, which also confirmed right-lateralization in the prefrontal region (see Figure 3D). The statistical significance can be seen in Table S2.

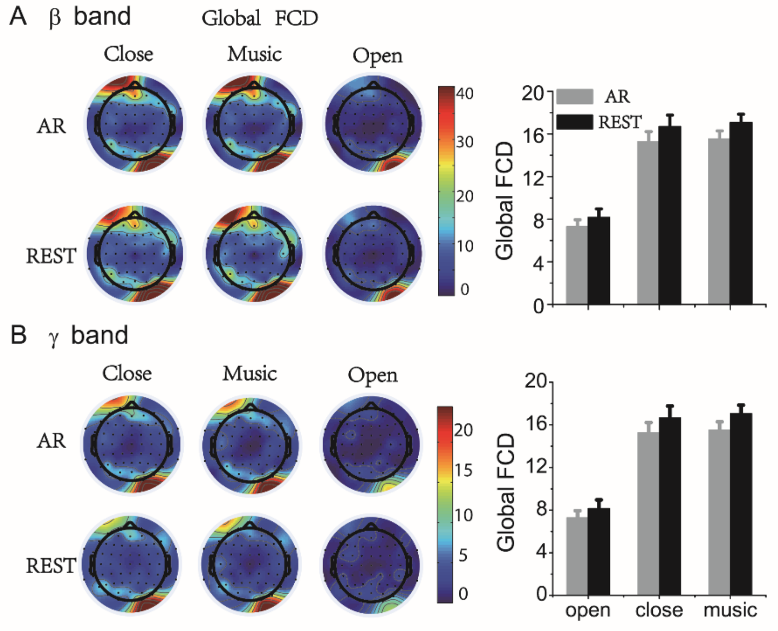

Figure S3

Local brain functional connectivity density (Local FCD) topography and average of Local FCD under AR and REST reference in high-frequency band situations. **(A)** Beta band. **(B)** Gamma band. The results of Local FCD were almost the same as for Global FCD. The statistical significance can be seen in Table S3.

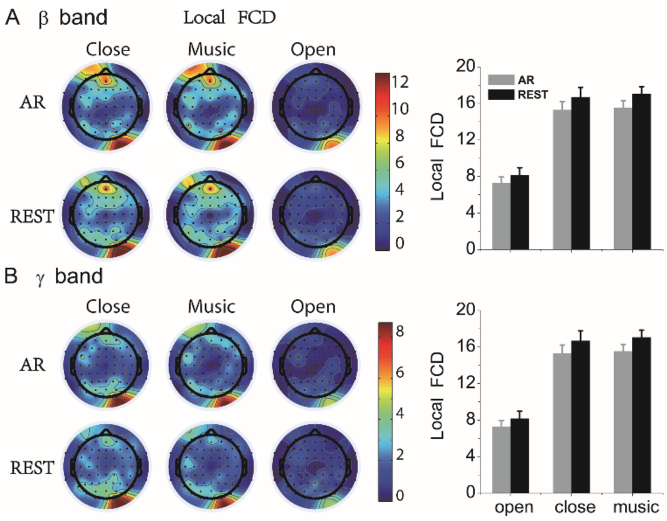

Figure S4

Statistical significance (Matlab ANOVA toolbox, ANOVA1, multcompare) of small-world characteristics among different states (eyes-open, eyes-closed and music) under REST and AR in all frequency band situations. **(A)** Full band. **(B)** Delta band. **(C)** Theta band. **(D)** Alpha band. **(E)** Beta band. **(F)** Gamma band. A p value < 0.05 indicates significant difference between different states. The difference between eyes-closed and eyes-open is significant in full, alpha, beta and gamma frequency bands. The difference between music-listening and eyes-open is significant in full, alpha, beta and gamma frequency bands, and in theta band under REST. There are no significant differences between music-listening and eyes-closed in all bands.

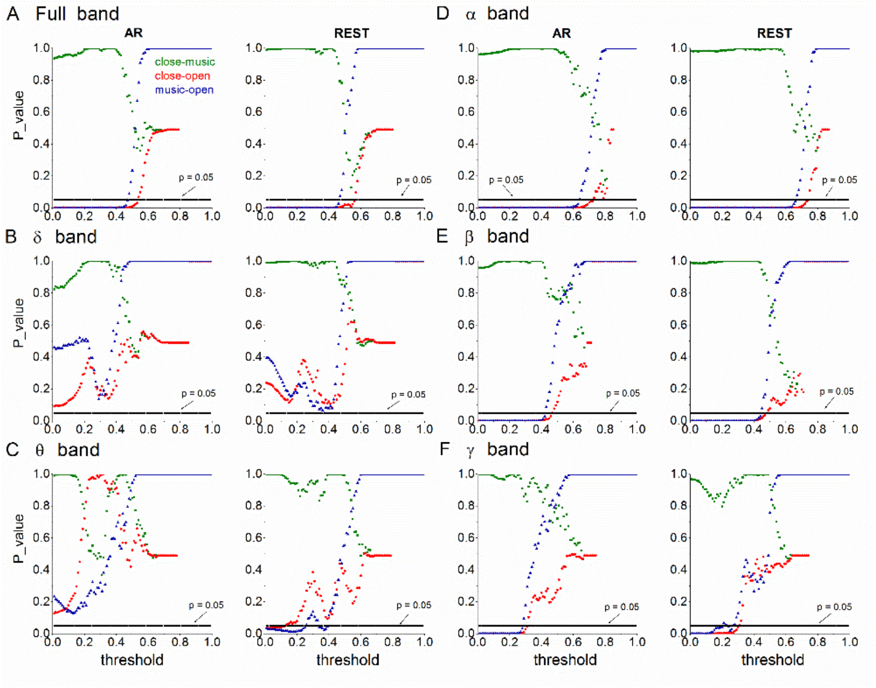

Table S1

Statistical significance (Matlab ANOVA toolbox, ANOVA1, multcompare) of the sum of the small-world effect in the threshold range from 0.2 to 0.5 among different states under REST and AR. The asterisk '*' represents p value < 0.05, which indicates significant difference, and the double asterisk '**' represents p value < 0.01, which indicates a greater significant difference.

		Closed - Music	Closed - Open	Music - Open
AR	Full	0.7812	9.0047e-07 (**)	2.4866e-10 (**)
	Delta	0.6148	0.0806	0.0157 (*)
	Theta	0.3285	0.3899	0.0095 (**)
	Alpha	0.8216	2.0544e-08 (**)	5.4648e-11 (**)
	Beta	0.7463	3.2482e-06 (**)	3.3617e-08 (**)
	Gamma	0.2581	0.0043 (**)	0.0037 (**)
REST	Full	0.9917	4.1202e-06 (**)	1.2637e-07 (**)
	Delta	0.9400	0.0292 (*)	9.6085e-04 (**)
	Theta	0.5526	0.0119 (*)	1.6227e-04 (**)
	Alpha	0.8965	4.4093e-09 (**)	2.6028e-12 (**)
	Beta	0.7761	3.7352e-07 (**)	2.5988e-09 (**)
	Gamma	0.4966	0.0019 (**)	0.0025 (**)

Table S2

Statistical significance (Matlab ANOVA toolbox, ANOVA1, multcompare) of the average of the global functional connectivity density (GFCD) among different states under REST and AR. The asterisk '*' represents p value < 0.05, which indicates significant difference, and the double asterisk '**' represents p value < 0.01, which indicates a greater significant difference.

		Close - Music	Close - Open	Music - Open
AR	Full	0.9211	3.7796e-09 (**)	4.7093e-09 (**)
	Delta	0.7813	0.0193 (*)	0.2030
	Theta	0.9338	0.0668	0.2444
	Alpha	0.9696	3.7732e-09 (**)	3.8959e-09 (**)
	Beta	0.9999	2.6659e-06 (**)	1.8475e-06 (**)
	Gamma	0.9869	0.0038 (**)	0.0118 (*)
REST	Full	0.9992	4.3532e-09 (**)	5.1554e-09 (**)
	Delta	0.9326	0.0213 (*)	0.1043
	Theta	1.0000	0.0304 (*)	0.0345 (*)
	Alpha	0.9963	3.7685e-09 (**)	3.7698e-09 (**)
	Beta	0.9979	2.1458e-07 (**)	7.7813e-08 (**)
	Gamma	0.8659	6.6713e-04 (**)	0.0111 (*)

Table S3

Statistical significance (Matlab ANOVA toolbox, ANOVA1, multcompare) of the average of the local functional connectivity density (LFCD) among different states under REST and AR. The asterisk '*' represents p value < 0.05, which indicates significant difference, and the double asterisk '**' represents p value < 0.01, which indicates a greater significant difference.

		Close - Music	Close - Open	Music - Open
AR	Full	0.9062	3.7717e-09 (**)	4.1987e-09 (**)
	Delta	0.7178	0.0164 (*)	0.2267
	Theta	0.9287	0.0377 (*)	0.1658
	Alpha	0.9802	3.7717e-09 (**)	3.8269e-09 (**)
	Beta	0.9926	1.4937e-06 (**)	3.3231e-07 (**)
	Gamma	0.9623	0.0056 (**)	0.0261 (*)
REST	Full	0.9972	4.8748e-09 (**)	7.7770e-09 (**)
	Delta	0.7900	0.0310 (*)	0.2663
	Theta	0.9991	0.0336 (*)	0.0226 (*)
	Alpha	0.9999	3.7683e-09 (**)	3.7684e-09 (**)
	Beta	0.9984	2.3727e-07 (**)	9.4549e-08 (**)
	Gamma	0.6538	0.0013 (**)	0.0550
